# A Longitudinal Study Examining Physical Activity Habit Formation

**DOI:** 10.3390/bs16040535

**Published:** 2026-04-02

**Authors:** Thomas McAlpine, Caitlin Liddelow, Jessica Charlesworth, Enrique Mergelsberg, Astrid Green, Elizaveta Novoradovskaya, Teagan Franz, Darren Haywood, Frank D. Baughman, Hayley Breare, Barbara Mullan

**Affiliations:** 1 enAble Institute, Faculty of Health Sciences, Curtin University, Perth, WA 6102, Australia; thomas.mcalpine@curtin.edu.au (T.M.);; 2 School of Population Health, Curtin University, Perth, WA 6102, Australia; 3 School of Human Sciences, University of Western Australia, Crawley, WA 6009, Australia; 4 Human Performance Research Centre, School of Sport, Exercise, and Rehabilitation, INSIGHT Research Institute, Faculty of Health, University of Technology Sydney (UTS), Sydney, NSW 2007, Australia; 5 Department of Mental Health, St Vincent’s Hospital Melbourne, Fitzroy, VIC 3065, Australia

**Keywords:** habit, automaticity, physical activity, cues, lockdown

## Abstract

Habits are important factors that help guide the performance of health behaviours, yet little is known about how habits form following a major context change. To observe the habit trajectory of physical activity engagement and assess the relationship between habit trajectory and theoretical determinants of habit formation, a longitudinal design was used to track physical activity habit trajectory over 12 weeks during COVID-19 lockdowns. Participants (N = 41) selected a cue related to physical activity, to assist in increasing their engagement. At baseline, and every two days, participants reported their behaviour, habit, and cue exposure. Trait self-control, history of physical activity behaviour, and demographic information were also collected at baseline. Physical activity habit strength significantly increased from baseline to the final assessment 12 weeks later; however substantial variability was observed in growth over time and neither the linear nor the logarithmic growth model provided a good fit at the overall level. Nonetheless, higher levels of self-control were significantly related to faster habit strength formation. Individual differences in habit formation require further investigation to better understand why some individuals form habits while others do not.

## 1. Introduction

Habit is fundamental to regular engagement in and maintenance of health behaviours, underpinning the automatic, context-driven performance of actions that sustain health and wellbeing over time (Kwasnicka et al., 2016; Mergelsberg et al., 2020). The distinction between behaviour and habit is pivotal; while behaviour may be consciously enacted, habit reflects the automaticity that develops through repeated action in a consistent context. This shift in focus, from the frequency of behaviour to the strength of habit, has highlighted that automaticity is the key determinant for sustaining long-term behaviour change, especially in domains such as physical activity (Gardner, 2015; Gardner et al., 2023).

According to habit theory, when behaviours are repeated in response to consistent contextual cues, they become increasingly automatic and efficient (Gardner, 2015; Lally et al., 2010; Mullan & Novoradovskaya, 2018). Habits, marked by their automaticity, demands less cognitive effort to perform the behaviour (Lally et al., 2010; Orbell & Verplanken, 2010). Reflective processes such as motivation and intention are typically important at the start of habit formation, but, over time and with repetition in stable contexts, behavioural initiation (the necessary preparatory behaviours leading up to the behaviour itself including the decision) becomes predominantly automatic (Hagger, 2019). This process is relevant even for complex (multi-step) behaviours like physical activity, where not every step is performed automatically, but the habit can still drive consistent initiation and execution (Gardner et al., 2020; Phillips & Mullan, 2023a, 2023b).

Research has identified a range of psychological factors that influence habit formation, including both reflective and automatic processes. Motivation and the strategic use of cues are especially significant (Fournier et al., 2017; Mergelsberg et al., 2020; Mullan et al., 2014), with cues facilitating the initiation and maintenance of habits (Booker & Mullan, 2013). For instance, Liddelow et al. (2021) found that contextual cues, like brushing teeth before taking medication, were the strongest predictors of medication adherence. Further, the formation of a new habit is most effective when the action is repeated in a stable context and in response to a cue or multiple cues (Keller et al., 2021; van der Weiden et al., 2020). Self-control, or the ability to regulate responses to internal and external stimuli (Baumeister et al., 2007) may also play a role; however, while some studies support its importance for physical activity habit formation (Gillebaart & Adriaanse, 2017; Phipps et al., 2025), others do not (van der Weiden et al., 2020). It may be that self-control is particularly relevant in the early stages of habit development and plays a less important role as automaticity increases (Hagger, 2019; Rhodes & Rebar, 2018). 

Longitudinal research is critical for understanding how habits form and evolve. Evidence suggests that it typically takes two to three months of repeated action in a stable context for habits to emerge, with habit strength tending to increase slowly at first before plateauing (Hagger, 2019; Keller et al., 2021; Lally et al., 2010; van der Weiden et al., 2020). The influential study by Lally et al. (2010) found that forming a habit took between 59 days and three months and followed a non-linear, asymptotic trajectory. However, despite its prominence, the Lally model has never been directly replicated, and its generalisability to the context of habits in real-world circumstances, particularly in the presence of environmental disruptions, remains untested. There is a clear need for studies that examine whether these findings hold in different contexts and populations.

Habits are strongly context dependent. When the physical or social context shifts, the automaticity underpinning habitual behaviours can be disrupted (Jager, 2003; Thomas et al., 2016). Even temporary changes in context can reduce cue exposure and, consequently, the likelihood of automatic behaviour initiation (Verplanken et al., 2008). On the other hand, such moments of disruption may create windows of opportunity for deliberate behaviour change and the development of new habits (Verplanken et al., 2018; Verplanken & Roy, 2016). However, how habit formation resumes after changes in the environment, and how this process compares to typical habit formation, is not well understood. This gap has major implications for helping individuals build or rebuild health behaviour habits, such as physical activity, when setbacks or disruptions occur (e.g., change of gym, altered work schedule, relocation to new city).

The COVID-19 pandemic provided a unique context for studying habits. Lockdowns and restrictions disrupted habits and routines worldwide, leading to reduced physical activity and negative impacts on wellbeing and quality of life (Adams-Prassl et al., 2020; Liddelow et al., 2023; Maltagliati et al., 2021; Richter & Heidinger, 2021; Stockwell et al., 2021). These widespread disruptions offered a rare window of opportunity to investigate the process of habit formation and re-formation under real-world conditions, and to clarify the roles of repetition, cues, and self-control when established habits are interrupted.

The importance of understanding habit formation is emphasised by the critical need to increase engagement in physical activity. Regular participation in physical activity provides numerous health benefits, including prevention of non-communicable diseases such as cardiovascular disease and diabetes (Tremblay et al., 2011; Warburton et al., 2006), and reductions in depression and anxiety (Saxena et al., 2005; Vella et al., 2023). Physical activity has even been shown to be as effective as medication for treating depression and depressive symptoms (Heissel et al., 2023), yet engagement remains low. In the United States, fewer than half of adults meet recommended aerobic activity guidelines, and only a quarter meet the guidelines for both aerobic and muscle-strengthening activity (Elgaddal et al., 2022). Further, high-income countries are seeing declining participation in physical activity over time (Guthold et al., 2018). Barriers to regular activity include practical constraints, such as time and money, as well as psychological factors like low self-efficacy and competence (Hoare et al., 2017; Reichert et al., 2007; Spinney & Millward, 2010; Teixeira et al., 2012). Traditionally, interventions have targeted motivational and self-regulatory processes but have shown mixed results for sustained change (Greaves et al., 2011). The emergence of habit-based interventions represents a promising avenue for supporting long-term increases in physical activity (Gardner et al., 2023).

### The Present Study

Building on the findings of Lally et al. (2010), Gardner et al. (2023), and Mergelsberg et al. (2020) the aim of the current study was to investigate the trajectories of the formation of a physical activity habit during a unique window of opportunity among people who wanted to increase their physical activity after experiencing notable change to their regular circumstances (i.e., during COVID-19 lockdown). In line with previous research, we hypothesised that;

**H1.**  *A logarithmic growth curve model would fit habit formation trajectories significantly better than a linear model (i.e., habit strength will grow faster to begin with before flattening out as it reaches a peak).*

Additionally, we aimed to understand the role that behavioural repetition, frequency of cue use, and trait self-control play in the habit formation trajectory over time. To address this aim, we hypothesised that:

**H2.**  *Behavioural repetition, frequency of cue use, and trait self-control will be associated with physical activity habit trajectories formed by participants. Specifically, people with higher levels across these variables would form a habit faster (e.g., people with higher levels of trait self-control would form a habit faster than those with low self-control).*

## 2. Materials and Methods

### 2.1. Design

A longitudinal within-participants design was used to track the habit trajectory of physical activity engagement. Habit was assessed every two days over a 12-week period, with a total of 43 time-points across the study period.

### 2.2. Participants

Participants were recruited using the online crowdsourcing platform, CloudResearch (Litman et al., 2017). The study was advertised between May and July 2020, when most states in the United States (US) were experiencing some degree of COVID-19 related lockdown restrictions. To be eligible to participate, participants had to reside in the US, be experiencing some changes to their daily lifestyle due to COVID-19 restrictions, indicate that they had decreased their physical activity while in COVID-19 lockdown, and indicate that they wanted to change this behaviour.

Participants were paid $3 USD for their time completing the baseline survey, then were paid incrementally for completing follow-up surveys each fortnight (i.e., $2 USD for the first two fortnights, $3 USD for the third fortnight, $4 USD for the fourth fortnight, $5 USD for the fifth, and $6 USD for the sixth). Participants also received a bonus of $10 USD if they completed 38 out of the 42 follow-up surveys. The maximum amount that participants could be paid was $35 USD over the 12-week study period.

### 2.3. Procedure

Ethics approval was obtained from Curtin University’s Human Ethics Committee (HRE2017-0173) and all participants provided informed consent before participating. Via an online survey, participants completed baseline measures which included demographics, past physical activity behaviour (before lockdown), current physical activity behaviour (during lockdown), habit strength, and trait self-control. To ensure participants’ understanding of the behaviour, physical activity was defined to participants as an activity “that got your heart racing faster and made you sweat, for longer than 30 min”, following recommendations from Centers for Disease Control and Prevention (2023). Participants were also asked to specify a single cue that would help them engage in/change their physical activity behaviour over the next 12 weeks. An explanation of what cues are, and some examples of cues were provided (e.g., exercising as soon as you/your partner wake up in the morning), before participants were instructed to *“spend the next few minutes thinking of a cue that you can match to physical activity, to assist you in changing your behavior”.* Participants were then instructed to write down their cue on a piece of paper and keep it somewhere safe so they could be reminded of the cue if they were to forget it. The baseline survey took approximately 15 min to complete. Participants then completed short follow-up online surveys every two days (42 in total), which included measures of behaviour, habit strength, and the use of their chosen cue. Each follow-up survey was only available for completion for 24 h and took approximately two minutes to complete.

### 2.4. Baseline Measures

#### 2.4.1. Self-Control

Trait self-control was measured using the Brief Self-Control Scale (Tangney et al., 2004), which consists of 13 items asking participants about their ability to control their thoughts, emotions and behaviours (e.g., “I am good at resisting temptation”). Response options ranged from 1 (not at all like me) to 5 (very much like me). Scores were summed, with higher scores indicating higher trait self-control. Internal consistency was high in this sample (α = 0.86).

#### 2.4.2. Past and Current Physical Activity Behaviour

Participants were asked, on average, how many times per week they engaged in physical activity that got their heart racing faster and made them sweat, for longer than 30 min. For pragmatic reasons, this single-item measure was selected to minimise burden so that it could be asked again at each follow-up, and was based off recommendations from the Centers for Disease Control and Prevention (2023). This was asked with respect to their average weekly physical activity performance both *before* and *since* COVID-19 restrictive measures. Answers were recorded on an 8-point slider scale, from 0 (never) to 7 (daily).

#### 2.4.3. Habit Strength

This was measured using the four item Self-Report Behavioural Automaticity Index (Gardner et al., 2012; Verplanken & Orbell, 2003). The stem “Engaging in physical activity that makes my heart beat faster and makes me sweat for longer than 30 min per day is something…” preceded four statements (e.g., “I do automatically”). Responses were recorded on a 7-point Likert scale from 1 (strongly disagree) to 7 (strongly agree). Scores were averaged for each behaviour, with higher scores indicating higher habit strength. Internal consistency for this measure at baseline was excellent (α = 0.96).

### 2.5. Follow-Up Measures

#### 2.5.1. Behavioural Repetition

Behaviour over the preceding two days was assessed with a single item: “Have you engaged in exercise that got your heart racing and made you sweat, for longer than 30 min over the last two days?”. Response options were “Yes” and “No”, coded as 1 and 0, respectively. Scores were summed to create a total behavioural repetition score. Higher scores indicated greater physical activity frequency.

#### 2.5.2. Frequency of Cue Use

Participants answered a single item: “Thinking back to the cue you specified in the baseline questionnaire; did you use this cue to help you increase your physical activity?”. Respondents answered ‘Yes’, ‘No’ or ‘Unsure’. The number of ‘Yes’ responses were summed to create a total cue frequency score.

#### 2.5.3. Habit Strength

As per baseline but assessed every two days.

### 2.6. Data Preparation and Analysis

Power for this study was calculated on the basis of a non-linear (logarithmic growth curve) model being able to outperform a linear model, as estimated by comparing model AIC when fit to sample data simulated from previous estimates (Lally et al., 2010). Power was taken as the proportion of times ΔAIC > 10 between models (in favour of the non-linear model), representing substantial support for the superior fitting model (Burnham & Anderson, 2004). The nlme (Pinheiro et al., 2021) and the simr (Green & MacLeod, 2016) packages were used in R (version 4.4) to simulate 100 datasets across each varying sample size ranging from 10 to 100, incremented by 10. For each simulation, the parameters in Lally et al.’s (2010) exponential growth model were used to generate data according to the formula $y=a-be^{-ct}$, where ‘a’ represents the asymptote, ‘b’ represents the difference between the asymptote and the starting value, ‘c’ represents the decay rate, and t is the day number. To reflect realistic variation and noise in the data, parameters were allowed to vary for each individual during simulation. Simulations showed that even with very small sample sizes (e.g., N = 10), power was well above 90%. To account for expected missing data across time points, we determined that if the effect sizes demonstrated by Lally et al. (2010) are reasonable estimates of the true parameters for habit growth formation over time, at least 20 participants would be required to detect these effects. See Supplementary Materials S1 for more information.

To address our first hypothesis, habit strength trajectories were modelled using a growth curve analysis of the lavaan package in *R* (Rosseel, 2012). This allows for the estimation of inter-individual variability in intra-individual patterns of change over time (Curran et al., 2010). We fit both a linear model and a non-linear growth model based on the parameters reported by Lally et al. (2010) and compared model fit using CFI (>0.90), TLI (>0.90), RMSEA (<0.08), −2Loglikelihood, and AIC. The Full Information Maximum Likelihood (FIML) methodology was chosen to deal with missing data (see below). This method estimates the missing value by determining the value that maximises the likelihood function based on the sample data.

To assess our second hypothesis, we selected the optimal null model as determined in hypothesis one and added covariates in two additional steps. The first step included demographic variables (age, gender, and lockdown status), and the second added the theory-informed variables of interest (behavioural repetition, frequency of cue use, and self-control). Model fit was assessed using the same criteria as in the first hypothesis.

### 2.7. Missing Data

Participant data were included if they completed 21 (50%) or more of the 42 follow-ups, as recommended by Newman (2003). Of 91 participants who initially signed up to participate, data for *n* = 41 (45%) participants were retained and analysed. There were no significant differences between those included and excluded in the final analyses based on demographics (all, *p* > 0.05), which included previous history of physical activity. For the other variables of interest, the only significant difference was found in ‘lockdown status’ where those excluded more frequently reported no lockdown measures in place (28.6%) compared to those included (7.3%; *p* = 0.021).

Given that the remaining number of participants provided less than the recommended proportion of timepoints—a ratio of at least 2:1 (Rosseel, 2012)—timepoints needed to be removed from the analysis to ensure the model was properly identified. To maintain the continuity of the trajectories as much as possible, we removed non-consecutive timepoints with the most missing data. Removed time points and slope loadings for each model can be seen in Supplementary Materials S2. Results from the simplified models will be presented where 24 time points were retained. Model fit was much poorer in the model with all timepoints (Supplementary Materials S3), although the parameter estimates were very similar^1^.

## 3. Results

Participants were between 24 and 72 years of age (*M* = 40.0 years, *SD* = 11.9 years). Further participant descriptive statistics can be found in Table 1. At baseline, participants reported they engaged in physical activity, on average, 1.67 days per week (*SD* = 1.86 days). When asked how frequently they engaged in physical activity prior to COVID-19 restrictions, participants reported higher physical activity at, on average, 2.76 days per week (*SD* = 1.68 days). Across timepoints the mean number of participants engaging in physical activity since previous assessment was 25.14 (64.95%; see Supplementary Materials S5 for full breakdown by time point).

**Figure 1:**
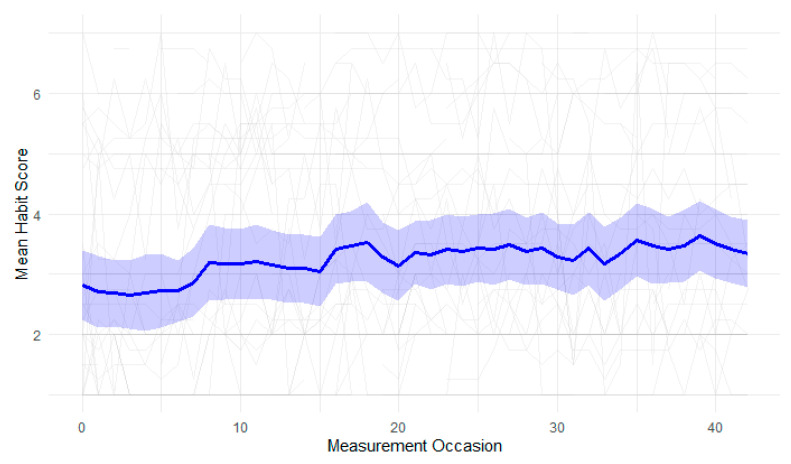
Individual habit strength trajectories of participants overlayed with mean habit strength and 95% confidence intervals across time.

Inspection of the complete data (see Figure 1) showed that physical activity habit strength increased minimally over time with substantial variability in the individual trajectories, despite a paired sample *t*-test indicating a significant increase from baseline (*M* = 2.82, *SD* = 1.83) to final assessment (*M* = 3.34, *SD* = 1.69), *t*(37) = 2.25, *p* = 0.019, two-tailed, Cohen’s *D* = 0.40. Consequently, neither the linear nor the logarithmic growth models fit the data well, and goodness-of-fit indices were all below acceptable thresholds (see Table 2).

Based on model fit alone, the linear model slightly outperformed the logarithmic model (Table 2), but significant variance in the intercept and slope indicated that not all participants started with the same baseline level of habit strength, nor increased their habit strength at the same rate.

We then tested the influence of the demographic and theoretical variables on the slope and intercept of the linear model in two separate steps to determine if this improved model fit to acceptable levels. Neither age, gender, nor lockdown status were significantly related to either the intercept or slope, and model fit did not increase substantially (CFI = 0.538, TLI = 0.555, Log Likelihood = −1030.59, AIC = 2131.18, RMSEA = 0.306). These were therefore dropped before entering the theoretical predictors in the final step. Model fit in the final step was similar, but marginally improved (CFI = 0.559, TLI = 0.575, Log Likelihood = −1022.90, AIC = 2115.81, RMSEA = 0.295).

The final model (see Table 3) showed that neither behaviour frequency, cue frequency, nor trait self-control were significantly related to starting values of habit strength. Furthermore, only self-control was positively and significantly related to the slope, indicating that those with higher levels of self-control improved habit over time more than those with lower levels. Nonetheless, the estimate for the slope was significantly less than zero in the final step, which suggested that habit strength decreased over time when behavioural frequency, cue frequency and self-control were all zero. As with the null model, the variance estimates for both the intercept and the slope were significant, which suggested that the theoretical predictors were unable to account for all the variance in trajectories. Interestingly, in the final step, the intercept and the slope negatively covaried, which suggested that after accounting for self-control, behavioural frequency, and cue frequency, those with weaker starting habits increased habit strength more over time.

## 4. Discussion

The aim of this research was to investigate the trajectories of the formation of a physical activity habit when people experience a context change (i.e., during COVID-19 lockdown). While average levels of habit strength increased significantly from baseline to the final timepoint, the results revealed considerable individual variability in habit strength trajectories, with neither linear nor logarithmic growth models providing a satisfactory fit to the data. Despite poor model fit, the variability in the trajectories is an important finding to discuss in and of itself. It suggests that the process of habit formation is complex and may not conform to a predictable pattern across all, or even the majority, of individuals, similarly observed by Baretta et al. (2025).

This is not uncommon in relation to habit formation, such that research has indicated that, despite participants being motivated to form a habit, only about half performed the behaviour enough to establish a habit (Judah et al., 2018; van der Weiden et al., 2020). An alternative explanation for the limited improvement in some participants may be due to the complexity of the behaviour examined, whereby exercising takes multiple steps to perform (e.g., preparing gym clothes, planning a gym routine, doing the gym routine). This can be conceptualised as consisting of numerous preparatory and enactment steps (Phillips & Mullan, 2023a) thereby providing greater opportunity for competing (e.g., less time consuming, lower effort, greater and more immediate gratification) to interrupt and disrupt the chain of actions required for full execution (Kaushal et al., 2025). Forming habits for simple (one-step) behaviours, such as turning off a light switch when leaving the room, is generally accepted as an easier process than for more complex (multi-step) behaviours like physical activity (Mullan & Novoradovskaya, 2018; Phillips & Mullan, 2023b). This research helps our understanding of how to improve habits for multi-step health behaviours with distal rewards but further investigation to better understand how best to help form habits for these behaviours is still needed.

When examining potential predictors of habit growth, demographic factors such as age, gender, and lockdown status were found to have no significant relationship with either the starting point (intercept) or the rate of change (slope) in habit strength. It is perhaps surprising that lockdown status was not related to either the intercept or the slope of habit strength, since differences in severity of the lockdown would seemingly influence the opportunity to exercise and therefore the ability to form physical activity habits. One explanation for this is that individuals who were advised to stay home may have adhered to guidelines as much as those with strict lockdown orders, ultimately experiencing similar conditions.

Theoretical variables, including behavioural frequency, cue frequency, and trait self-control, also did not significantly predict initial habit strength. This contrasts with previous research which indicated the importance of behavioural repetition and frequency of cue use for both triggering habitual behaviour (Neal et al., 2012; Phillips & Gardner, 2016), and forming habitual behaviour (Booker & Mullan, 2013; Keller et al., 2021). A potential explanation for the difference in findings, might lie in the difficulty of forming a physical activity habit in new circumstances, reflected by the small, albeit significant overall increase in habit strength in the present study.

Trait self-control stood out as the only variable positively and significantly related to the slope. This should be cautiously interpreted given the poor overall model fit; however results suggest that individuals with higher levels of self-control tended to increase their physical activity habits more effectively over time. Self-control has been intricately tied to habitual processes to facilitate engagement in health behaviours (Kothe et al., 2015), including exercise (De Bruijn et al., 2012; Phipps et al., 2025). This is particularly true for health behaviours where rewards are more distal in nature, such as physical activity where numerous benefits such as weight loss (Swift et al., 2018), and reduced risk of disease (Kyu et al., 2016) are not observed until the long term. It may be that for these multi-step behaviours; self-control can help individuals to engage in the behaviour long enough for it to become habitual. Consequently, physical activity interventions may seek to target aspects of self-control to help individuals begin to form habits for physical activity. Despite this, the overall model fit after accounting for self-control remained only marginally improved, and substantial unexplained variance persisted in both the intercept and slope, suggesting that other unmeasured factors may influence habit formation trajectories.

Notably, the final step of the analysis revealed a negative covariance between the intercept and slope, meaning that after accounting for self-control, behavioural frequency, and cue frequency, participants who started with weaker habits tended to show greater improvements over time. This finding adds an important dimension to our understanding of habit formation, indicating that individuals with lower baseline habit strength are not necessarily disadvantaged in their ability to develop stronger habits; in fact, they may experience more pronounced growth.

The present results highlight the challenge of modelling habit formation at the group level, given the marked individual variability in both starting points and rates of change and suggests that group-level aggregate models may be insufficient to capture the highly idiosyncratic nature of habit formation following a major context change. The failure of both logarithmic and linear models to sufficiently capture the data structure means that the timing of peak habit strength remains indeterminate for this sample, unlike previous research which obtained estimates of how long it takes to form a habit (Lally et al., 2010; Singh et al., 2024). It should be noted however, that the variability in both the shape and length of time of the habit formation process was acknowledged in these works too. For example, Lally et al. (2010) report subsets of people where the general model did not fit well, and Singh et al. (2024) describe variability between 4 and 335 days in terms of mean or median times to form a habit.

Altogether, these findings draw attention to the importance of considering individual differences and the limitations of relying on aggregate models to predict behaviour change. As highlighted by Wen et al. (2025), variability in physical activity habits is a robust phenomenon, and future research should account for this diversity when designing interventions and theoretical models. For example, future research should employ more flexible, person-centred analytic approaches, such as latent class growth modelling (Muthén & Muthén, 2000), which may better account for heterogeneity in habit trajectories and offer valuable insights into subgroups with distinct trajectories (e.g., stable strong habits, habit formation, habit degradation; Rebar et al., 2022). Alternatively, research could focus on distinguishing between habits related to the instigation and execution of the behaviour and observing these separately. Additionally, further investigation into individual characteristics—beyond self-control and the demographic and theoretical predictors assessed here—could help clarify the mechanisms underlying successful habit formation, particularly in contexts of discontinuity or change. For instance, factors such as motivation, mood and affect may be particularly important for physical activity habits (Hopkins et al., 2022). Ultimately, a better understanding of the heterogeneity in habit growth patterns will be crucial for tailoring interventions and advancing theory in the domain of physical activity habits.

### Strengths and Limitations

The present findings are novel in that they extend beyond current understandings and suggest that higher levels of self-control may contribute to faster habit formation, and not just that it is an important factor for habits to form at all. However, to better understand why some people form habits, and others do not, future research is needed to help identify other individual differences (e.g., perceived reward) which are potentially important in habit formation following disruption to regular routines, particularly in the context of other health behaviours.

The longitudinal 12-week study period, with multiple time points each week, also allowed for the more naturalistic observation of the physical activity habit formation process with frequent assessments allowing for more accurate recall of behaviour, cue use, and habit strength. Yet, given participant attrition, not all timepoints were able to be used. This means that although the included timepoints in the final models were selected for practical reasons (i.e., lowest proportion of missing data, and to ensure a good representation of timepoints across the 12 weeks), there is still a risk that these selected points did not fully represent the habit formation process. Nonetheless, sensitivity analyses showed the same pattern of results using the complement subset of timepoints, providing some confidence that results reported here were reflective of the sample.

Moreover, it is likely that the multi-stepped and difficult nature of physical activity performance (Hagger, 2019), contributed to the limited overall improvement in habit strength and even longer periods of observation might be required to observe true habit formation without more active intervention (e.g., 106–154 days; Singh et al., 2024). Aside from asking participants to select a cue to assist with habit formation, participants were not provided with any education or any other active support to help guide formation, which are both important predictors of physical activity habits (Craig et al., 2025; Juwita et al., 2025). Future research in physical activity habit formation therefore should consider providing more active and deliberate support throughout the development phase, which would allow for a more pronounced growth in habit strength amenable to model testing.

The process by which participants selected their cues in the present study should also be considered. While the eligibility criteria and study instructions around the cue use were framed in a way to encourage a novel physical activity habit to be formed, the online nature made it difficult to guarantee this was the case for all participants. Furthermore, different types of cues (e.g., time-based, location based) are differentially related to physical activity habit strength (Pimm et al., 2016) and although outside the scope of the present study, the role of cue type could be considered in future work to maximise the likelihood of habit formation.

Another limitation of the current study was the lack of use of a validated physical activity behaviour measure in favour of a brief binary measure of physical activity engagement. Although the measure needed to be short due to the frequency in which they were provided to participants, it is possible that the limited sensitivity of this measure in capturing meaningful variation in true behaviour could have contributed to the null findings observed for the relationship between behavioural repetition and habit strength. Despite the observation of habit strength being the primary focus of the study, future studies should still consider capturing physical activity behaviour in more detail (e.g., the types of physical activity engaged in, as well as time of day and duration) to better understand the context surrounding engagement in physical activity and the impact this might have on habit formation. Alternatively, objective measures such as accelerometry could be used to obtain physical activity data over consecutive days with minimal participant burden (Skender et al., 2016), which would eliminate recall bias, reduce measurement error, and allow for a more granular assessment of physical activity performance.

## 5. Conclusions

This study is the first to demonstrate the inter-individual variance in physical activity habit formation under contextual changes. Findings from the current study are important as they demonstrate that the same simple habit-based interventions may not be successful for everyone, especially for multi-step behaviours such as physical activity. Additionally, trait self-control may be implicated in the speed in which physical activity habits are formed. However, future research is needed to replicate the findings of the current study to better understand the inter-individual variance among physical activity habit formation, but also in other habitual behaviour following a context change. This has important implications for designing effective interventions targeting habit formation for changing or maintaining health behaviours over time.

## Figures and Tables

**Table 1:** Participant characteristics (N = 41).

Characteristic	n	%
**Gender**		
Male	28	68.3
Female	13	31.7
Other	0	0
**Ethnicity**		
Caucasian	27	65.9
Hispanic or Latin	2	4.9
African American	5	12.2
Asian	3	7.3
European	3	7.3
Other	1	2.4
**Job before COVID-19**		
Employed full-time	30	73.2
Employed part-time	3	7.3
Employed on casual basis	0	0
Employed—working from home	4	9.8
Unemployed	4	9.8
Full-time student	0	0
**Current employment status**		
No change, still go to work	8	19.5
Reduced hours, still go to work	3	7.3
Work from home full time	16	39
Reduced hours, work from home	4	9.8
Still work casual hours	1	2.4
Full-time student online	0	0
Dropped to part-time online studies	0	0
Dropped studies completely	0	0
Lost my job, unemployed	1	2.4
Other	8	19.5
**Living situation**		
Live alone	7	17.1
With housemates/friends	2	4.9
With partner/spouse	13	31.7
With family, including children	17	41.5
With elderly relatives	2	4.9
**Lockdown situation in state of residence**		
Business as usual	3	7.3
Advised not to leave the house	20	48.8
Strict lockdown	18	43.9
**Time staying at home since lockdown**		
One week or less	0	0
One or two weeks	1	2.4
More than two weeks	0	0
More than three weeks	35	85.4
Not applicable to me	5	12.2

**Table 2:** Model fit and estimates of the linear model compared to the logarithmic growth model.

	Linear			Logarithmic		
CFI	0.563			0.559		
TLI	0.591			0.587		
LL	−1032.931			−1033.983		
AIC	2123.862			2125.966		
RMSEA	0.322			0.324		
	Estimate	SE	z-value	Estimate	SE	z-value
Intercept	2.987	0.264	10.989 ***	2.113	0.334	6.333 ***
Slope	0.018	0.006	3.206 ***	0.035	0.009	3.857 ***
Variance Inter.	2.762	0.630	4.387 ***	4.278	1.011	4.233 ***
Variance Slope	0.001	0.000	4.218 ***	0.003	0.001	4.161 ***
Covariances I~~S	−0.016	0.010	−1.677	−0.080	0.024	−3.288 ***

*Note.* *** *p* < 0.001. I~~S = covariance between the intercept and the slope.

**Table 3:** Linear growth modelling results across all three steps (N = 41).

**Step 1 (Null Model)**	**Estimate**	**SE**	**z-value**
Intercept	2.90	0.26	10.99 ***
Slope	0.02	0.01	3.21 ***
Variance Inter.	2.76	0.63	4.39 ***
Variance Slope	0.00	0.00	4.22 ***
Covariances I~~S	−0.02	0.01	−1.68
**Step 2 (Demographic Predictors)**	**Estimate**	**SE**	**z-value**
Intercept			
Age	0.01	0.03	0.37
Gender	0.48	0.61	0.79
Lockdown	0.37	0.44	0.85
Slope			
Age	0.00	0.00	0.80
Gender	−0.00	0.01	−0.24
Lockdown	−0.02	0.01	−1.64
Intercept	1.00	1.34	0.75
Slope	0.04	0.03	1.44
Variance Inter.	2.62	0.60	4.38 ***
Variance Slope	0.00	0.00	4.12 ***
Covariances I~~S	−0.02	0.01	−1.65
**Step 3 (Theoretical Predictors)**	**Estimate**	**SE**	**z-value**
Intercept			
Beh. Freq.	0.01	0.04	1.32
Cue Freq.	0.02	0.03	0.67
Self-control	0.03	0.03	1.32
Slope			
Beh. Freq.	0.00	0.00	0.39
Cue Freq.	0.00	0.00	0.39
Self-control	0.01	0.00	2.38 *
Intercept	0.78	1.22	0.64
Slope	−0.05	0.02	−2.11 *
Variance Inter.	2.50	0.57	4.38 ***
Variance Slope	0.00	0.00	4.15 ***
Covariances I~~S	−0.02	0.01	−2.62 **

*Note.* * *p* < 0.05. ** *p* < 0.01. *** *p* < 0.001. I~~S = covariance between the intercept and the slope.

## Data Availability

The data that support the findings of this study are available on request from the corresponding author. The data are not publicly available due to privacy or ethical restrictions.

## Note

1 We also repeated models (Supplementary Materials S4) for main results presented here with the complement subset of timepoints and pattern of results were very similar.

## Supplementary Materials

The following supporting information can be downloaded at: https://www.mdpi.com/article/10.3390/bs16040535/s1, Table S1. Model Parameters and Standard Deviation for Simulation. Figure S1. Example Simulations Data for N = 10, 50, and 0.100; Table S2. Results of Power Analyses Across Sample Sizes; Figure S2. Difference in Model Fit with Increasing Sample Size; Table S3. Final Timepoints selected in the Growth Curve Analyses; Table S4. Baseline Model Fit and Estimates. Table S5. Baseline Model Fit and Estimates. Table S6. Frequency and Proportion of Physical Activity Behavioural by Timepoint.
