# Supplementary material for: A Longitudinal Study Examining Physical Activity Habit Formation
Source: Behav Sci (Basel). 2026 Apr 2;16(4):535. doi: 10.3390/bs16040535 (PMC13114073; doi:10.3390/bs16040535)
Supplement: Supplementary file 1 [file behavsci-16-00535-s001.zip › behavsci-4025046-supplementary.pdf]

Supplementary Material S1: Power Analyses

Power for this study was calculated on the basis of a non-linear (logarithmic growth curve) model being able to outperform a linear model, as estimated by comparing model AIC when fit on to sample data simulated from previous estimates (Lally et al., 2010). Power was taken as the proportion of times ΔAIC > 10 between models (in favour of the non-linear model), representing substantial support for the superior fitting model (Burnham & Anderson, 2004). The nlme (Pinheiro et al., 2021) and the simr (Green & MacLeod, 2016) packages were used in R (version 4.4) to simulate 100 datasets across each varying sample size ranging from 10-100, incremented by 10. For each simulation, the parameters in Lally et al.'s (2010) exponential growth model were used to generate data according to the formula $y = a - be^{-ct}$, where 'a' represents the asymptote, 'b' represents the difference between the asymptote and the starting value, 'c' represents the decay rate, and t is the day number. To reflect realistic variation and noise in the data, parameters were allowed to vary for each individual during simulation. Parameter values and standard deviations are shown in Supplementary Table S1 below.

Supplementary Table S1. *Model Parameters and Standard Deviation for Simulation*

Parameter	Parameter Value	Standard Deviation
a	35	8
b	30	8
c	0.042	0.02
t	43	N/A

Example data for the simulations across differing sample sizes are shown below in Figure S1.

Supplementary Figure S1. *Example Simulations Data for N = 10, 50, and 100.*

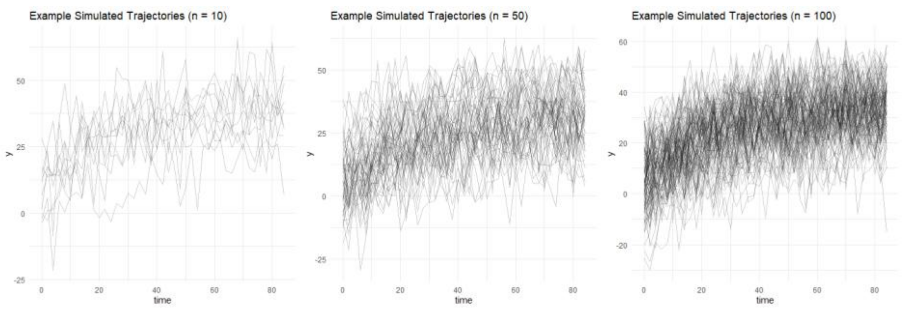

Simulations showed that even with very small sample sizes (e.g., N = 10), the non-linear model was preferred 100% of the time (see Table S2).

Supplementary Table S2. *Results of Power Analyses Across Sample Sizes*

Sample Size	Observed Power
10	1.00
20	0.99
30	1.00
40	1.00
50	1.00
60	0.99
70	1.00
80	1.00

90	1.00
100	1.00

Note. Power was calculated as the proportion of times in which the non-linear growth model fit the simulated data better than the linear model (as indicated by ΔAIC > 10).

Further plots of the AIC comparing each model across the different sample sizes were generated to observe model fit behaviour, and results show that model fit for the non-linear model over the linear model increases with sample size.

Supplementary Figure S2. *Difference in Model Fit with Increasing Sample Size*

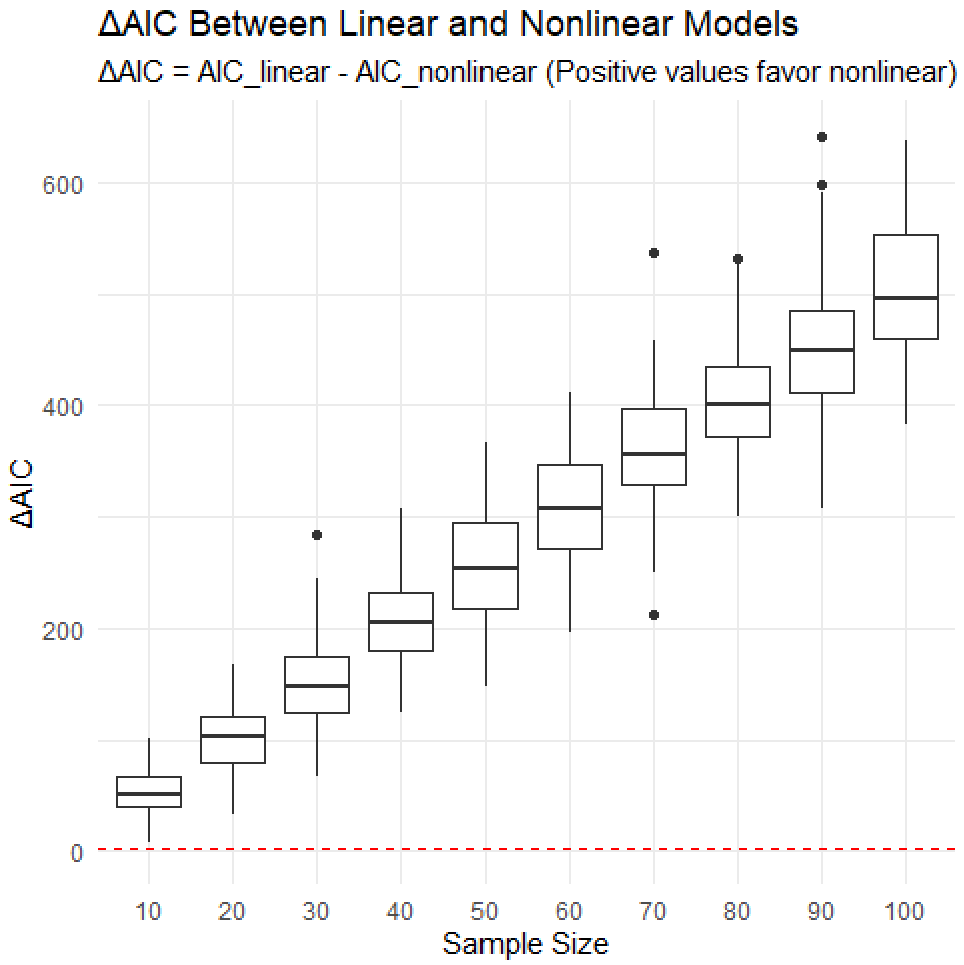

To account for expected missing data across time points, we determined that if the effect sizes demonstrated by Lally et al. (2010) are reasonable estimates of the true parameters for habit growth formation over time, at least 20 participants would be required to detect these effects.

Supplementary Material S2: Selection of Timepoints for Inclusion in Growth Models

Supplementary Table S3. *Final Timepoints selected in the Growth Curve Analyses*

Time Point	Included	Linear Slope Loading	Exponential Slope Loading
0 (Baseline)	Yes	0	0.00
1		1	7.74
2	Yes	2	12.27
3	Yes	3	15.48
4		4	17.97
5	Yes	5	20.01
6	Yes	6	21.73
7	Yes	7	23.22
8		8	24.54
9	Yes	9	25.71
10	Yes	10	26.78
11		11	27.75
12	Yes	12	28.64
13		13	29.47
14	Yes	14	30.24
15		15	30.96
16	Yes	16	31.64
17	Yes	17	32.28
18		18	32.88
19	Yes	19	33.45
20		20	34.00
21	Yes	21	34.52
22		22	35.01
23	Yes	23	35.49
24		24	35.94
25	Yes	25	36.38
26		26	36.80

27	Yes	27	37.21
28		28	37.60
29	Yes	29	37.98
30		30	38.35
31	Yes	31	38.70
32		32	39.04
33	Yes	33	39.38
34		34	39.70
35	Yes	35	40.02
36		36	40.32
37	Yes	37	40.62
38		38	40.91
39	Yes	39	41.19
40		40	41.47
41	Yes	41	41.74
42		42	42.00

Supplementary Material S3: Results Contrasting Simplified Model (24 timepoints) Against the Maximum Model (39 time points)

Supplementary Table S4. *Baseline Model Fit and Estimates*

	Simplified Model (k = 24)			*Full model (k =39)**		
**Linear Model**						
CFI	.563			.330		
TLI	.591			.357		
LL	-1032.93			-1641		
AIC	2123.862			3378.92		
RMSEA	.322			.405		
	Estimate	*SE*	*z-value*	*Estimate*	*SE*	*z-value*
Intercept	2.90	0.26	10.99***	2.94	0.273	10.78***
Slope	0.018	0.006	3.21***	0.016	0.006	2.91**
Variance Inter.	2.76	0.63	4.39***	3.01	0.67	4.45***
Variance Slope	0.001	.000	4.22***	0.001	0.000	4.36***
Covariances I~~S	-0.016	0.01	-1.68	-0.022	.011	-2.11*
**Logarithmic Model**						
CFI	0.559			.366		
TLI	.587			.394		
LL	-1033.98			-1544.59		
AIC	2125.97			3177.18		
RMSEA	0.324			0.389		
	Estimate	*SE*	*z-value*	*Estimate*	*SE*	*z-value*
Intercept	2.11	0.33	6.33***	2.23	0.34	6.56***
Slope	0.035	0.009	3.86***	0.032	0.009	3.60***

Variance Inter.	4.23	1.01	4.23***	4.48	1.06	4.24***
Variance Slope	0.003	0.001	4.161***	0.003	.001	4,24***
Covariances I~~S	-0.080	0.024	-3.29***	-0.081	0.024	-3.34***

* For the exp model this was without T4, T18, T15, T8 as the highest missing timepoints in order to get number of observations > k

Supplementary Material S4: Results Contrasting Simplified Model (24 timepoints) Against the Complement Model (Opposite subset of time points)

Supplementary Table S5. *Baseline Model Fit and Estimates*

	Simplified Model (k = 24)			*Complement model (k = 20)*		
**Linear Model (Null)**						
CFI	.563			.555		
TLI	.591			.587		
LL	-1032.93			-844.72		
AIC	2123.862			1739.44		
RMSEA	.322			.337		
	Estimate	*SE*	*z-value*	*Estimate*	*SE*	*z-value*
Intercept	2.90	0.26	10.99***	2.95	0.312	9.44***
Slope	0.018	0.006	3.21***	0.017	0.006	2.49*
Variance Inter.	2.76	0.63	4.39***	3.63	0.86	4.23***
Variance Slope	0.001	.000	4.22***	0.002	0.000	4.13***
Covariances I~~S	-0.016	0.01	-1.68	-0.038	.015	-2.55*
**Linear Model (Step 2)**						
Intercept						
Age	0.01	0.03	0.37	0.01	0.03	0.35
Gender	0.48	0.61	0.79	0.58	0.69	0.84
Lockdown	0.37	0.44	0.85	0.29	0.50	0.58
Slope						
Age	0.00	0.00	0.80	0.00	0.00	0.65
Gender	-0.00	0.01	-0.24	-0.01	0.02	-0.41
Lockdown	-0.02	0.01	-1.64	-0.01	0.01	-0.93

Intercept	1.00	1.34	0.75	1.10	1.52	0.73
Slope	0.04	0.03	1.44	0.03	0.03	0.96
Variance Inter.	2.62	0.60	4.38***	3.37	0.77	4.37***
Variance Slope	0.00	0.00	4.12***	0.002	0.000	4.21***
Covariances I~~S	0.02	0.01	-1.65	-0.035	0.01	-2.64**
**Linear Model (Step 3)**						
Intercept						
Beh. Freq.	0.01	0.04	1.32	0.02	0.04	0.39
Cue Freq.	0.02	0.03	0.67	0.02	0.03	0.47
Self Control	0.03	0.03	1.32	0.02	0.03	0.72
Slope						
Beh. Freq.	0.00	0.00	0.39	0.00	0.00	0.14
Cue Freq.	0.00	0.00	0.39	0.00	0.00	0.43
Self Control	0.01	0.00	2.38*	0.002	0.001	2.60**
Intercept	0.78	1.22	0.64	1.24	1.39	0.90
Slope	-0.05	0.02	-2.11*	-0.07	0.03	-2.34
Variance Inter.	2.50	0.57	4.38***	3.26	0.75	4.36***
Variance Slope	0.00	0.00	4.15***	0.001	0.000	4.16***
Covariances I~~S	-0.03	0.01	-2.62**	-0.04	0.01	-3.27***

Supplementary Material S5: Physical Activity Behaviour Repetition

Supplementary Table S6. Frequency and Proportion of Physical Activity Behavioural by Timepoint

Time Point	Behavioural Frequency
1	17 (49%)
2	26 (65%)
3	24 (62%)
4	20 (61%)
5	26 (67%)
6	29 (71%)
7	27 (69%)
8	24 (67%)
9	26 (70%)
10	28 (70%)
11	26 (70%)
12	27 (66%)
13	26 (65%)
14	30 (75%)
15	23 (66%)
16	28 (70%)
17	32 (78%)
18	19 (56%)
19	24 (60%)
20	26 (67%)
21	18 (45%)
22	26 (70%)
23	26 (63%)
24	24 (63%)
25	30 (71%)
26	24 (62%)
27	24 (60%)
28	27 (69%)
29	27 (68%)
30	23 (59%)
31	25 (61%)
32	24 (65%)
33	22 (58%)
34	25 (68%)
35	23 (61%)
36	27 (73%)
37	25 (61%)
38	21 (55%)
39	26 (67%)
40	28 (70%)
41	27 (68%)
42	26 (67%)
Mean	25 (65%)

Note. Percentages are based off number of complete responses reported for that time point.
